# Pancreatic Damage and Radiological Changes in Patients With COVID-19

**DOI:** 10.7759/cureus.14992

**Published:** 2021-05-12

**Authors:** Ahmet Bozdag, Yesim Eroglu, Ayse Sagmak Tartar, Pinar Gundogan Bozdag, Serpil Aglamis

**Affiliations:** 1 General Surgery, Firat University, Elazig, TUR; 2 Department of Radiology, Firat University School of Medicine, Elazig, TUR; 3 Department of Infectious Diseases and Clinical Microbiology, Firat University School of Medicine, Elazig, TUR; 4 Department of Radiology, Health Sciences University, Elazig Fethi Sekin City Hospital, Elazig, TUR

**Keywords:** pancreas, pancreatitis, covid-19, tomography, pancreatic ınjury

## Abstract

Background and objective

The novel coronavirus disease 2019 (COVID-19) primarily affects the lungs. However, others organs are also affected in varying degrees. We aimed to investigate the changes in pancreatic density on CT and its correlation with amylase/lipase values in patients diagnosed with COVID-19.

Materials and methods

Radiological changes using non-contrast CT and amylase/lipase values were evaluated retrospectively in patients admitted to the pandemic clinic. The patients were classified into two groups: [polymerase chain reaction (PCR)-positive and PCR-negative]. The correlation and difference between the data were evaluated statistically.

Results

There was no significant difference with respect to age and gender between the two groups (PCR-positive and PCR-negative). There was a significant difference in the head, neck, trunk, and tail of the pancreas and mean density values, but no statistically significant difference in amylase and lipase values between the two groups. No significant correlation was found using Spearman’s correlation test.

Conclusion

Based on our findings, pancreatic involvement and severe necrotizing pancreatitis can be seen in COVID-19 patients. Pancreatic involvement is more common in patients with severe disease. Patients with gastrointestinal complaints should be evaluated for pancreatitis and their amylase/lipase values should be assessed. We believe that decreased pancreatic density on CT scans can be an early sign of pancreatitis.

## Introduction

The novel coronavirus disease 2019 (COVID-19) is a viral disease that emerged in Wuhan, China in December 2019; it has subsequently spread all over the world and has been declared a global pandemic by the World Health Organization. It frequently affects the lungs and presents as pneumonia and respiratory failure. However, the heart, gastrointestinal system, and liver are also affected to varying degrees. In their study, Spinelli et al. reported COVID-19 patients having gastrointestinal symptoms similar to surgical diseases; in particular, they described the emergence of an acute pancreatitis-like scenario [1]. Other studies supporting the possibility of pancreatic damage due to COVID-19 soon followed [2-5]. Since the publication of these studies, there have been reports on cases of acute pancreatitis related to COVID-19 without additional pathology in the etiology [6-12].

The clinical diagnosis of acute pancreatitis requires the presence of at least two of the three revised Atlanta criteria. These are typical abdominal pain, a ≥3-fold increase in serum amylase and/or lipase activity, and characteristic findings on CT or other imaging modalities [13,14]. Most studies on COVID-19 in the literature have measured the amylase and lipase levels, but there are only a few studies related to radiological findings. In this study, we aimed to compare pancreatic density and laboratory findings of patients diagnosed with COVID-19 with those of a control group.

## Materials and methods

**Figure 1:**
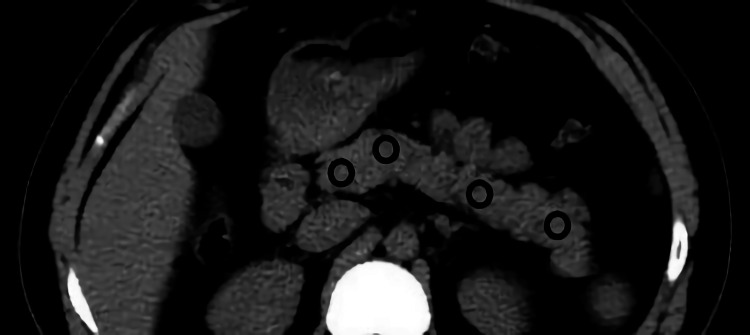
Measurement Measurement of the pancreatic head, neck, corpus, and tail densities by placing a circular region of interest on non-contrast axial CT images CT: computed tomography

We included patients who presented to the Fırat University Hospital's pandemic outpatient clinic between March 2020 and July 2020 with suspected COVID-19 and underwent a CT scan, and whose pancreatic tissue was present in the imaging area. The subjects were classified into two groups; group 1 included 59 polymerase chain reaction (PCR)-positive patients and group 2 included 42 PCR-negative patients. The findings of biochemical and radiological examinations performed at first admission were evaluated retrospectively using the hospital’s data system. Pancreatic density was calculated for each patient as Hounsfield units (HU) by considering an average 0.5 cm^2^ region of interest (ROI) in the head, neck, corpus, and tail sections of the pancreas on non-contrast CT images of each patient. For excluding the partial volume effect of peripancreatic fat tissue, care was taken not to make measurements from the pancreatic periphery. Additionally, no measurements were made from vascular structures (Figure 1). Amylase and lipase values of the patients were also recorded. Necessary permissions were obtained from the Firat University Non-Interventional Research Ethics Committee and the Ministry of Health (date: 01/10/2020; approval number: 2020/13-02).

All statistical analyses were performed using SPSS Statistics version 20.0 (IBM, Armonk, NY). The suitability of quantitative data for normal distribution was evaluated by the Shapiro-Wilk test. Because the quantitative data did not meet parametric assumptions, statistical comparison between the two independent groups was performed using the Mann-Whitney U test. Since the data showed a nonparametric distribution, descriptive statistics were presented as median (min-max). Spearman’s correlation coefficient method was used to evaluate the correlation between two quantitative independent variables that did not meet parametric assumptions. While qualitative data were analyzed using Pearson’s chi-square test, descriptive statistics were presented as frequency and percentage. The significance level was set at 0.05 for all tests.

## Results

Of the patients included in the study, group 1 had 26 (44.1%) females and 33 (55.9%) males whereas group 2 had 12 (28.6%) females and 30 (71.4%) males. There was no significant difference between the groups in terms of gender (p=0.113). The mean age of the patients [median (min-max)] in group 1 was [44 (18-82)] years and that in group 2 was [39 (22-89)] years. No significant difference was found between the groups in terms of age (p=0.491). Pancreatic density was calculated by taking an average of all four regions of the pancreas. Statistical comparison of the findings and descriptive statistics are given in Table 1. There was a significant difference between the groups in all four pancreatic regions and average pancreatic density. However, no statistically significant difference was found in the amylase and lipase values of the two groups. Additionally, correlation analysis between biochemical and radiological data revealed no statistically significant correlation.

**Table 1:** Statistical comparison of groups and descriptive statistics

Variables	Group 1, median (min–max), n=59	Group 2, median (min–max), n=42	P-value
Head density, HU	42 (10–57)	47.5 (28–67)	0.003
Neck density, HU	43 (15–59)	47 (28–62)	0.002
Corpus density, HU	42 (10–55)	49 (28–60)	<0.001
Tail density, HU	42 (12–53)	47 (28–61)	<0.001
Average density, HU	42 (11.75–55.25)	47.87 (28–62.5)	<0.001
Amylase, U/L	61 (34–795)	67 (32–419)	0.878
Lipase, U/L	46 (24–2,971)	30 (18–595)	0.074

## Discussion

The new type of coronavirus disease was first identified in December 2019 and has since turned into a global pandemic and public health issue. An article published in March 2020 stated that an acute pancreatitis-like scenario can be observed in COVID-19 patients. Since then, further investigations have been conducted on pancreatic damage in COVID-19 patients, resulting in the publication of numerous case reports [1,2,6,7].

Similar to other viruses from the “Coronaviridae” family, such as the severe acute respiratory syndrome (SARS) and the Middle East respiratory syndrome (MERS) viruses, the severe acute respiratory syndrome coronavirus 2 (SARS-CoV-2) primarily affects the respiratory system. Additionally, it affects the liver, kidneys, heart, and gastrointestinal organs. These viruses enter the human cells using angiotensin-converting enzyme 2 (ACE2) receptors. Liu et al. found that SARS-CoV-2 was present more in the pancreas than in the lungs of normal individuals and reported that the virus was expressed in both exocrine glands and endocrine islets of the pancreas. This observation has been accepted to be the basic mechanism of pancreatic damage caused by COVID-19 [4]. Apart from this, increased systemic inflammation and microvascular thrombosis are also believed to play a role in pancreatic damage [6,15].

In COVID-19 patients, pancreatic damage can be observed in approximately 1-2% of mild cases and 17% of severe cases [3]. In addition, recurrent acute pancreatitis has been reported in one case [12]. However, in the articles published so far, the diagnosis was made only on the basis of the elevation of amylase and lipase levels. These articles have been criticized because this elevation can be caused by various reasons other than acute pancreatitis [16]. Radiological findings have been presented only in case reports, wherein necrotizing pancreatitis has been reported in one patient [9]. In this study, we radiologically evaluated the increase in pancreas' size, streaking in peripancreatic adipose tissue, and changes in pancreatic density, which are included in the Atlanta criteria [13,14].

**Figure 2:**
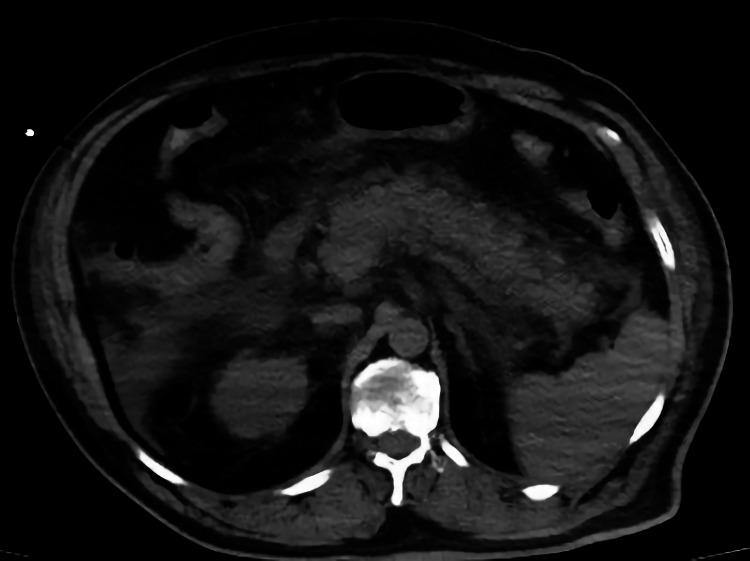
Increase in the size of the pancreas Non-contrast axial CT image showing an increase in pancreas' size CT: computed tomography

**Figure 3:**
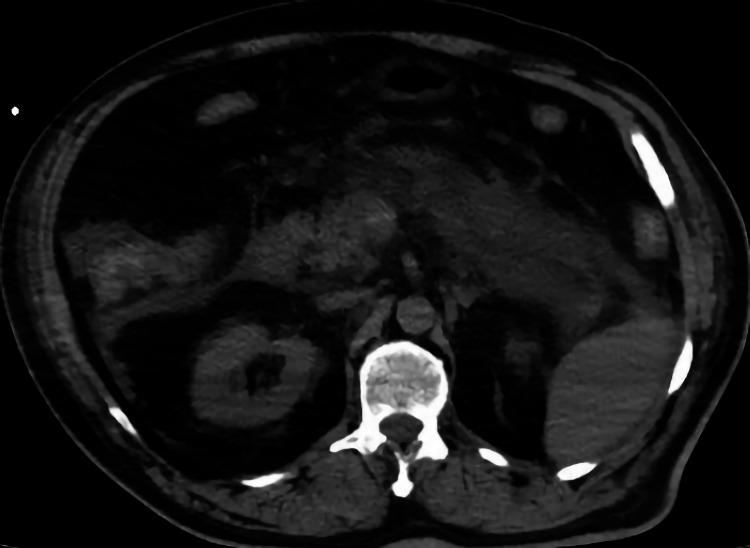
Increase in peripancreatic streaking, and fluid Non-contrast axial CT image showing an increase in peripancreatic streaking, and fluid CT: computed tomography

In our study, more than a three-fold increase in amylase and lipase was detected in four patients, two in group 1 and two in group 2. Of these, the two patients in group 1 also experienced abdominal pain. Typical signs of edematous pancreatitis (pancreatic size increase, peripancreatic streaking, and fluid) were detected in one of the patients (Figures 2, 3). This patient eventually died due to multiple organ failures. The pancreatic density of all pancreatic sections was decreased in COVID-19 patients compared to the controls. No additional radiological findings were observed in other patients. We think that the absence of additional radiological findings is due to the admission of the patients to the hospital in the early period of the infection. In an autopsy study, parenchymal cell degeneration and necrosis in the pancreas, hyaline thrombus formation in small vessels, and pathological changes similar to those in chronic diseases were observed, but no evidence of coronavirus infection was found in these organs [15].

There are some limitations to our study. Primarily, it was a retrospective study and only the data registered in the patient information system could be accessed. Also, our measurements were made from non-contrast imaging. The fact that the patients were evaluated before any treatment was initiated eliminated the chance to observe any differences caused by antiviral drugs.

Viral pancreatitis has been well described in the literature, and it is most commonly caused by mumps virus, measles virus, coxsackievirus, Epstein-Barr virus, and hepatitis B virus [17,18]. After the respiratory system, COVID-19 most commonly affects the gastrointestinal system. Hence, the most common symptom in patients after fever, dyspnea, and cough is abdominal pain. In addition, patients presenting with only gastrointestinal complaints have been reported in the literature. High amylase and lipase levels are frequently encountered in these patients [19].

In our opinion, laboratory parameters must be examined for pancreatitis in COVID-19 patients presenting with gastrointestinal complaints and greater care must be taken in treating them. We believe that pancreatic density measurement using CT can be used in the early diagnosis of pancreatitis in COVID-19 patients.

## Conclusions

There are still many unknowns about COVID-19. Since the pancreas is a tissue containing ACE2 receptors, it is particularly affected in severe COVID-19 disease. A decrease in density on CT is an early finding suggestive of pancreatic involvement in these patients. The decrease in pancreatic density is very significant in terms of determining the treatment and follow-up of the patients, especially patients with widespread pulmonary involvement at the time of initial diagnosis.
